# An assessment of the benefit-risk balance of FOLFIRINOX in metastatic pancreatic adenocarcinoma

**DOI:** 10.18632/oncotarget.12761

**Published:** 2016-10-19

**Authors:** Julien Péron, Pascal Roy, Thierry Conroy, Françoise Desseigne, Marc Ychou, Sophie Gourgou-Bourgade, Trevor Stanbury, Laurent Roche, Brice Ozenne, Marc Buyse

**Affiliations:** ^1^ CNRS, UMR 5558, Laboratoire de Biométrie et Biologie Evolutive, Equipe Biostatistique-Santé, Service de Biostatistiques, Centre Hospitalier Lyon-Sud, Hospices Civils de Lyon, 69495 Pierre-Bénite, France; ^2^ Medical Oncology Department, Centre Hospitalier Lyon-Sud, Institut de Cancérologie des Hospices Civils de Lyon-IC-HCL, 69495 Pierre-Bénite, France; ^3^ Institut de Cancérologie de Lorraine, Alexis Vautrin Center, 54500 Vandœuvre-lès-Nancy, France; ^4^ Medical Oncology Department, Centre Léon Bérard, 69373 Lyon, France; ^5^ Institut Régional du Cancer Montpellier, Val d'Aurelle, 34298 Montpellier, France; ^6^ R&D UNICANCER, 75013 Paris, France; ^7^ International Drug Development Institute (IDDI), San Francisco, CA 94109, USA

**Keywords:** multi-criteria analysis, pancreatic cancer, benefit-risk assessment, randomized controlled trial, statistics as topic

## Abstract

**Background:**

We sought to assess the benefit-risk balance of FOLFIRINOX versus gemcitabine in patients with metastatic pancreatic adenocarcinoma.

**Methods:**

We used generalized pairwise comparisons. This statistical method permits the simultaneous analysis of several prioritized outcome measures. The first priority outcome was survival time (OS). Differences in OS that exceeded two months were considered clinically relevant. The second priority outcome was toxicity. The overall treatment effect was quantified using the net chance of a better outcome, which can be interpreted as the net probability for a random patient treated in the FOLFIRINOX group to have a better overall outcome than a random patient in the gemcitabine group.

**Results:**

In this trial 342 patients received either FOLFIRINOX or gemcitabine. The net chance of a better outcome favored strongly and significantly the FOLFIRINOX group (24.7; P<.001), suggesting a favorable benefit-risk balance of FOLFIRINOX versus gemcitabine. The positive benefit-risk balance of FOLFIRINOX was observed throughout all sensitivity analyses.

**Conclusions:**

Generalized pairwise comparisons are useful to perform a quantitative assessment of the benefit-risk balance of new treatments. It provides a clinically intuitive way of comparing patients with respect to all important efficacy and toxicity outcomes. Overall the benefit-risk balance of FOLFIRINOX was strongly positive.

## INTRODUCTION

Efficacy and safety are the primary considerations when characterizing a treatment effect. Both US Food and Drug Administration and the European Medicines Agency have stressed the importance of a more structured and transparent approach to benefit–risk assessment (BRA) in the evaluation of new therapies [1, 2]. In oncology clinical trials, efficacy and safety outcomes are usually analyzed and reported independently [3, 4].

Patients with metastatic pancreatic cancer have a poor prognosis and the historical first line regimen is gemcitabine [5]. Several new systemic therapies have been investigated in combination with gemcitabine in randomized trials. Among them the NCIC Clinical Trials Group Study PA.3 phase III trial investigated the addition of erlotinib to gemcitabine. Both overall survival (OS) and progression-free survival (PFS) were significantly better for the combination treatment [6]. However a benefit-risk assessment was performed using generalized pairwise comparison and was not in favor of adding erlotinib to gemcitabine for the treatment of patients with advanced pancreatic cancer [7].

In the last few years, two chemotherapy combination regimens have shown in randomized trials to improve largely patients' outcomes. FOLFIRINOX (5-fluorouracil, oxaliplatin, irinotecan, leucovorin) has shown superiority over gemcitabine in both PFS (hazard ratio for disease progression, 0.47; 95% CI, 0.37 to 0.59; P<0.001) and OS (hazard ratio for death, 0.57; 95% confidence interval [CI], 0.45 to 0.73; P<0.001) [8]. However, there is controversy as to whether the survival benefits of the FOLFIRINOX combination outweigh the associated toxicities [9]. More recently, a trial comparing a combination of nab-paclitaxel and gemcitabine versus gemcitabine alone demonstrated a statistically significant survival benefit for this new doublet, introducing another option for the management of advanced pancreatic cancer [10]. With the introduction of these therapeutic options it was of interest to compute an assessment of the benefit-risk balance of FOLFIRINOX. We report here such an assessment based on the method of generalized pairwise comparisons [11, 12]. This method extends the Mann-Whitney-Wilcoxon test for several prioritized outcome and in the presence of censored data. It allows one to calculate and test the overall effect of a new treatment based on any number of prioritized outcomes, some reflecting benefit from the intervention (e.g., survival or time to progression), and the others reflecting harms (e.g., treatment-related toxicities and side effects).

## RESULTS

### Efficacy outcome

The main analysis of efficacy and safety was conducted after 273 deaths (126 in the FOLFIRINOX group and 147 in the gemcitabine group) and has already been reported [8]. Overall survival was significantly longer in the FOLFIRINOX group with an estimated HR of 0.57 (95% CI, 0.45 to 0.72; P<0·001; log-rank test stratified for performance status and primary tumor localization). Median survival times were 11.1 months in the FOLFIRINOX group versus versus 6.7 months in the gemcitabine group.

Three hundred and seventeen patients had developed progressive disease or had died at the end of the trial. Progression-free survival was significantly longer in the FOLFIRINOX group with an estimated HR of 0·47 (95% CI, 0·37 to 0·59; P<0·001; median, 6.3 months versus 3.2 months).

### Toxicity outcomes

The frequency grade ≥ 3 treatment-related clinical AEs was higher for the FOLFIRINOX group (69%) compared with the gemcitabine group (60%), but the difference was not statistically significant (P=0.083) (Table 1). The increase in grade ≥ 3 AEs was especially notable for the neurologic adverse events (11.1% versus 2.3%, P=0.0028), gastrointestinal adverse events (33.9% versus 24.6%, P=0.042), infectious adverse events (10.5% versus 5.3%, P=0.096), and general adverse events (28.7% versus 22.8%, P=0.19).

**Table 1:** Worst grade toxicity by treatment group

Worst grade related AE	FOLFIRINOX group (n=171)	Gemcitabine group (n=171)
**Grade 0**	6 (3.5%)	2 (1.2%)
**Grade 1**	7 (4.1%)	5 (2.9%)
**Grade 2**	40 (23.4%)	62 (36.3%)
**Grade 3**	81 (47.4%)	67 (39.2%)
**Grade 4**	36 (21.1%)	34 (19.9%)
**Grade 5**	1 (0.6%)	1 (0.6%)

AE = Adverse Events

### Benefit-risk assessment

The net chance of a better overall survival in the FOLFIRINOX group (first priority outcome with a threshold of clinical significance at 2 months) was 27.4%, 95% CI, 14.2% to 40.6% (thus favoring FOLFIRINOX), and the net chance of a better toxicity (second priority outcome) was −2.7 (thus favoring gemcitabine) among patients neutral on the OS outcome. The net chance of a better overall outcome favored significantly the FOLFIRINOX group (Overall Δ[FOLFIRINOX] = 24.7%, 95% CI, 11.1% to 38.0%; P<.001), suggesting a favorable benefit-risk balance of FOLFIRINOX versus gemcitabine (Table 2).

**Table 2:** Main analysis of the benefit-risk balance of FOFLIRINOX versus gemcitabine

Priority	Pairwise probabilities (%)		Δ[FOLFIRINOX]
	FOLFIRINOX > Gemcitabine	Gemcitabine > FOLFIRINOX	
**1 : OS (threshold = 2 months)**	54.4%	26.9%	27.4%
**2 : Worst related AE grade**	4.8%	7.5%	−2.7%
**Overall**	59.2%	34.4%	**24.7% (P<.001)**

OS = Overall Survival; AE = Adverse Events; Δ[FOLFIRINOX] = Net chance of a better outcome in the FOLFIRINOX group.

### Sensitivity analyses

**Figure 1:**
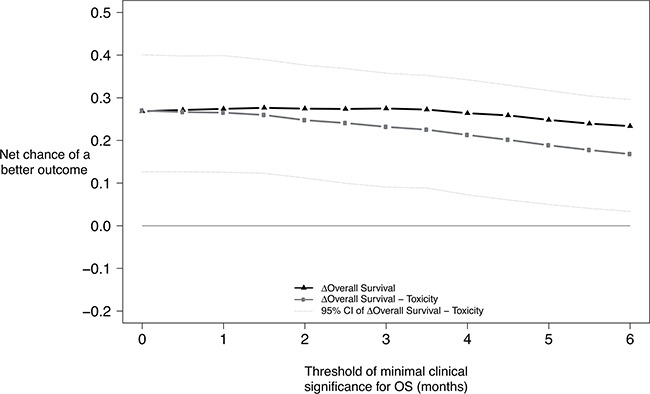
Benefit-risk of FOLFIRINOX according to the minimum survival benefit considered clinically significant Net chance of a better outcome with FOLFIRINOX according to the minimum survival benefit considered clinically meaningful. First priority outcome: Overall survival. Second priority outcome: Worst grade of at least possibly related adverse events. Solid black line with asterisks : Net chance of a better survival with. Solid light-grey line with points : Nat chance of a better overall outcome with FOLFIRINOX.

**Figure 2:**
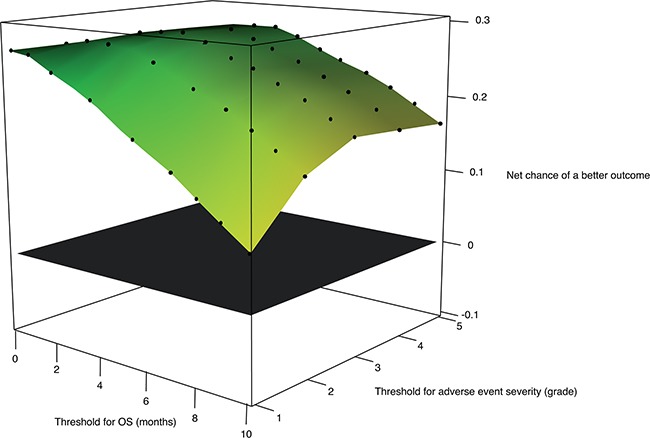
Benefit-risk of FOLFIRINOX according to the survival threshold of clinical significance and to the adverse event severity threshold (in number of adverse event grades) Net chance of a better overall outcome with FOLFIRINOX according to the minimum survival benefit considered clinically significant and to the minimal difference in adverse event grade considered clinically significant. First priority outcome: Overall survival. Second priority outcome: Worst grade of at least possibly related adverse events. In green, the net chance of a better overall outcome is strongly in favor of FOLFIRINOX. In yellow the net chance of a better overall outcome is mildly in favor of FOLFIRINOX. The plane in black indicates no treatment benefit.

The analysis was repeated with various values for OS threshold, varying between 0 and 6 months. When the OS threshold was set at 0 month, meaning that any difference in OS was considered clinically significant, the overall analysis was statistically significant (net chance of a better overall outcome with FOLFIRINOX = 27.0%, 95% CI, 12.7% to 40.1%; P<.001). Even when only differences in OS larger than 6 months were considered clinically significant (threshold for OS = 6 months), the benefit-risk balance favored significantly the FOLFIRINOX group (net chance of a better overall outcome with FOLFIRINOX = 16.8%, 95% CI, 3.4% to 29.6%; P=.013) (Figure 1 and Table A in the Appendix). Figure 2 extends Figure 1 with an additional sensitivity analysis for increasing thresholds for treatment-related clinical AEs; this figure shows that FOLFIRINOX is uniformly better regardless of the thresholds chosen.

When related AEs were considered as a binary outcome (occurrence of at least one grade ≥ 3 related adverse event versus no grade ≥ 3 related adverse event), the net chance of a better overall outcome favored significantly the FOLFIRINOX group (25.3%, 95% CI, 11.8% to 38.8%; P<.001, Table B, in the Appendix).

When biological adverse events were included in the overall analysis of the benefit-risk balance, the net chance of a better overall outcome with FOLFIRINOX varied only slightly (24.2, 95% CI, −10.7 to 37.6; P<0.001).

Comprehensive sensitivity analyses of the benefit-risk were carried out using various thresholds for OS, and worst adverse event grade. Some scenarios with clinically meaningful choices of endpoint prioritization and of thresholds are presented in Table 3. All the scenarios considered favored the FOLFIRINOX group in term of benefit-risk balance.

**Table 3:** Further sensitivity analyses of the benefit-risk balance FOFLIRINOX versus gemcitabine, using several prioritized endpoints and several threshold values for the outcomes of interest

Priority	Threshold	Pairwise probabilities		Δ[FOLFIRINOX]
		FOLFIRINOX > Gemcitabine	FOLFIRINOX > Gemcitabine	
**1 : OS**	9 months	26.1%	7.7%	18.4%
**3 : Worst related AE grade**	3 grades	2.3%	1.2%	1.1%
**4 : OS**	6 months	11.2%	6.1%	5.1%
**6 : Worst related AE grade**	2 grades	2.7%	4.7%	−2.0%
**7 : OS**	3 months	10.3%	6.3%	4.0%
**9 : Worst related AE grade**	1 grade	4.2%	8.1%	−3.9%
**Overall**		56.8%	34.2%	**22.6% (P<.001)**
**1 : Worst related AE grade**	3 grades	3.2%	1.8%	1.4%
**2 : OS**	6 months	36.1%	13.3%	22.8%
**3 : PFS**	6 months	4.6%	1.1%	3.5%
**4 : Worst related AE grade**	2 grades	2.5%	4.0%	−1.5%
**5 : OS**	3 months	8.3%	5.5%	2.8%
**6 : PFS**	3 months	3.2%	1.2%	2.0%
**7 : Worst related AE grade**	1 grade	3.1%	5.4%	−2.3%
**8 : OS**	0 months	3.2%	3.3%	−0.1%
**9 : PFS**	0 months	0.0%	0.0%	0.0%
**Overall**		64.3%	35.7%	**28.6% (P<.001)**

AE = Adverse Events; OS = Overall Survival; PFS = Progression Free Survival; Δ[FOLFIRINOX] = Net chance of a better outcome in the FOLFIRINOX group.

## DISCUSSION

We have used generalized pairwise comparisons using several outcomes to perform an assessment of the benefit-risk balance of FOLFIRINOX versus gemcitabine for first-line treatment of patients with metastatic pancreatic cancer. The main analysis of the benefit-risk balance, as well as all the sensitivity analyses, was strongly in favor of the FOLFIRINOX.

A similar analysis of the benefit-risk balance was previously conducted on the NCIC PA.3 phase III trial. This trial investigated the addition of erlotinib to gemcitabine in patients with advanced pancreatic cancer [6]. Both survival and progression-free survival were significantly better in the erlotinib group, but the overall benefits were of modest magnitude. The benefit-risk balance, assessed withgeneralized comparison [11] was not in favor of the erlotinib.

More recently, the treatment options for metastatic pancreatic adenocarcinomas have increased with the approval of nab-paclitaxel (albumin-bound paclitaxel) as first line therapy in combination with gemcitabine [10]. In the absence of head-to-head clinical trials comparing FOLFIRINOX and the combination of nab-paclitaxel and gemcitabine, the best treatment approach to use for untreated metastatic pancreatic adenocarcinoma is not known. We believe that the two regimens should be compared in terms of benefit-risk balance, because of their different toxicity profiles. The assessment of the benefit risk balance of new treatments evaluated in randomized trials has several important limitations. First, the methods used for collection, and analysis of adverse events are heterogeneous [7]. Using AE rate as a measure of treatment toxicity could then lead to different conclusions in different trials according to the methods used. Then, other factors have the potential to impair patients' quality of lifeFor example the use of a portable pump, the number of visit to the hospital, the use of additional therapies such as antibiotics or granulocyte colony-stimulating factor, the length of adverse events, have relevance in the overall assessment of a treatment. These factors were not included in the benefit-risk balance analysis reported in this manuscript. However they could theoretically be included in an overall analysis of the treatment effect using generalized pairwise comparison. In fact, all relevant factors can be included in the analysis as long as they are reproducibly and precisely collected.

One advantage of generalized pairwise comparison in the assessment of the benefit-risk balance is that it gives higher priority to the outcome considered clinically more important. The method can analyze simultaneously any number of outcomes. Each prioritized outcome is associated with a threshold of clinical relevance, and as such it reflects the thinking process of patients, clinicians and decision makers, who try to assess the net effect of a new treatment on several outcomes considered to be of clinical importance. Moreover, a single outcome can be included repeatedly at several priorities with different thresholds values.

Other methods have been proposed to help the scientific assessment of the benefit-risk balance of interventions [2]. QALY is a measurement of survival that assigns a weight in each period of time according to the quality of life of this period [13]. It might be used to adjust a gain in survival to an increased level of toxicity by assigning a smallest weight to the time of survival with significant toxicity. However it requires clearly defined health states, as well as weights for each state, which might be difficult to establish when planning a trial. The use of QALY as a primary endpoint in clinical trials has been limited for this reason. QALY is often considered more suited for medico-economic evaluation [14]. Overall Treatment Utility (OTU) can be used to combine subjective and objective measures of the treatment effect into a single composite endpoint. However the respective importance of the different treatment effects included in OTU may be difficult to understand and to report [15].

When assessing the benefit-risk balance with generalized pairwise comparisons, sensitivity analyses are useful to assess the robustness of the main analysis conclusion. Indeed, the conclusion may rest entirely on arbitrary (though arguably relevant) choices made regarding outcome priorities and thresholds values. Most clinicians and patients would agree that small gains in survival cannot be considered as a positive outcome if such gains are obtained at the expense of severe toxicities. However, the minimal survival benefit threshold for which most patients would accept to experience a treatment-related adverse event is often unknown. Investigators can now use generalized pairwise comparisons to test the benefit-risk balance of investigational therapies, depending on the level of tolerable toxicity that is deemed acceptable for a given magnitude of survival benefit. That is the purpose of the sensitivity analyses reported in Figure 1 and Table 3. Each scenario reported in these analyses could be chosen as the most relevant scenario by investigators or patients, depending on their expectation on a treatment efficacy and their tolerance to adverse events. Throughout all the scenarios, the benefit-risk balance favored the FOLFIRINOX group. In other trials investigating other regimens, the sensitivity analyses might lean to opposite conclusions. In such case, generalized pairwise comparisons could be used to help clinicians and patients in the choice of the best treatment depending on the patient own expectations of the treatment effect.

Generalized pairwise comparisons are useful to perform a quantitative assessment of the benefit-risk balance of a new treatment as compared with a standard therapy. It provides a clinically intuitive way of comparing patients with respect to all important efficacy and toxicity outcomes, with full flexibility as to the priority of each outcome, and a threshold of clinical significance. The benefit-risk balance of FOLFIRINOX versus gemcitabine in the Prodige 4 - ACCORD 11/0402 trial was positive.

## MATERIALS AND METHODS

### Overview

The Prodige 4 - ACCORD 11/0402 trial (NCT00112658) randomized patients with metastatic pancreatic cancer to a combination chemotherapy regimen consisting of oxaliplatin, irinotecan, fluorouracil, and leucovorin (FOLFIRINOX) as compared with gemcitabine as first-line therapy. The primary outcome was OS. PFS and toxicity were secondary outcomes.

In this trial, 342 patients were stratified according to center, performance status (0 vs. 1), and primary tumor localization (the head vs. the body or tail of the pancreas), and randomly assigned in a 1:1 ratio to receive FOLFIRINOX or gemcitabine. Progression was evaluated using Response Evaluation Criteria in Solid Tumors (V1.0) every 2 months. Toxicity was assessed at every visit using the National Cancer Institute Common Toxicity Criteria version 3.0.

### Generalized pairwise comparisons

We applied generalized pairwise comparisons extended to several outcome measures (a benefit outcome, and a risk outcome). A full description of the method has been previously published [11]. Briefly, pairwise comparisons require consideration of all possible pairs of patients, one taken from the FOLFIRINOX group, and the other taken from the gemcitabine group. Pairwise comparisons are stratified for the stratification factors used in the randomization process.

The outcomes of these two patients are compared according to the first priority outcome. The pair is said to be ‘favorable’ if the outcome of the patient in the FOLFIRINOX group is better than the outcome of the patient in the gemcitabine group and ‘unfavorable’ if the outcome of the patient in the FOLFIRINOX group is worse than the outcome of the patient in the gemcitabine group. The pair is said to be ‘uninformative’ if it cannot be determined which of the two patients has a better outcome (e.g., because of censoring, or because of missing data), and to be ‘neutral’ when the two observations are equal, or when the difference of outcomes does not reach a pre-specified threshold of clinical significance. When a pair is uninformative on a survival outcome as a result of censoring, the probabilities for this pair to be favorable, unfavorable, or neutral are calculated from the time to the censored observations, and from the Kaplan-Meier estimates of the survival functions [16]. Such a pairwise comparison is carried out for all pairs of patients, and the difference between the probability for a pair to be favorable and the probability to be unfavorable pairs is calculated for the first priority outcome. This difference is called the net chance of a better outcome for the first priority outcome [17, 18].

For pairwise comparisons that are neutral or uninformative for the first priority outcome, the second priority outcome is used in turn to classify the pair as favorable, unfavorable, neutral, or uninformative (Table 4). After consideration of the second priority outcome, the “net chance of a better overall outcome” is calculated to provide an overall assessment of both the benefit and the risks of the treatment, suitably prioritized.

**Table 4:** Generalized pairwise comparisons for two prioritized outcomes

First priority outcome	Second priority outcome	Pair is
favorable	ignored	favorable
unfavorable	ignored	unfavorable
neutral/uninformative	favorable	favorable
neutral/uninformative	unfavorable	unfavorable
neutral/uninformative	neutral/uninformative	neutral/uninformative

### Standard analysis of efficacy and toxicity

A log-rank test stratified for stratification factors at baseline was used to compare treatment groups in terms of survival. For each patient, the worst grade of adverse event at least possibly related to the study treatment (“treatment-related AEs”) observed during the duration of the study was recorded. The incidence of worst grade adverse events were compared using a stratified chi-square test. Biological adverse events were excluded from the overall analysis of the treatment toxicity. All analyses were performed on all randomly assigned patients as per the intent-to-treat principle.

### Main analysis of the benefit-risk balance

The first priority outcome used in the main analysis was OS. Only differences in OS exceeding two months were considered clinically significant. The second priority outcome was treatment-related AEs, with patients experiencing the lower AE grade considered to have had a more favorable outcome. Treatment groups were compared using the net chance of a better outcome with FOLFIRINOX over gemcitabine (Δ[FOLFIRINOX]). A randomization test stratified by performance status and extent of disease at diagnosis was performed to test the null hypothesis (*H*_0_ : Δ[FOLFIRINOX] = 0). The contribution of each outcome to Δ[FOLFIRINOX] was calculated.

### Sensitivity analyses

The impact of the choice of outcomes, thresholds, and priority on the results was assessed in sensitivity analyses. First, the main analysis was repeated with various thresholds for the minimal OS difference considered as clinically significant, ranging from 0 (any difference in OS considered clinically meaningful) to 6 months. Second, the toxicity outcome was defined as a binary variable where only grade ≥ 3 AEs were considered. Finally, a wide range of scenarios integrating OS, PFS, and AE grades with several successive thresholds were built in order to provide a comprehensive assessment of the treatment effects. For each scenario, the net chance of a better overall outcome in the FOLFIRINOX group was estimated and tested for statistical significance.

### Statistical analyses

All analyses reported in this manuscript were performed using the extended procedure of generalized comparisons, using the method “Peron” of the package BuyseTest in the R software (available on CRAN) [16].

## APPENDIX TABLES


