# Supplementary material for: An assessment of the benefit-risk balance of FOLFIRINOX in metastatic pancreatic adenocarcinoma
Source: Oncotarget. 2016 Oct 19;7(50):82953–60. doi: 10.18632/oncotarget.12761 (PMC5347744; doi:10.18632/oncotarget.12761)
Supplement: Supplementary file 1 [file oncotarget-07-82953-s001.pdf]

An assessment of the benefit-risk balance of FOLFIRINOX in metastatic pancreatic adenocarcinoma

APPENDIX TABLES

Appendix Table A: Sensitivity analysis of the benefit-risk balance of FOFLIRINOX versus gemcitabine

Δ[FOLFIRINOX] (95%CI)					
**Threshold for adverse event severity (grade)**					
**Threshold for OS (months)**	**1**	**2**	**3**	**4**	**5**
0 month	27.0% (12.7-40.1)	26.9% (12.7-40.2)	26.9% (12.6-40.1)	26.9% (12.6-40.1)	26.5% (12.5-40.1)
2 months	24.7% (11.2-37.7)	27.4% (14.1-40.5)	28.1% (15.1-41.2)	27.9% (14.7-40.9)	27.4% (14.0-40.4)
4 months	21.3% (7.2-34.2)	25.7% (13.0-38.0)	27.4% (14.8-39.6)	26.9% (14.4-39.0)	26.4% (13.8-38.5)
6 months	16.8% (3.4-29.6)	22.4% (10.0-33.3)	24.5% (12.9-35.4)	23.9% (12.7-34.7)	23.3% (12.3-34.5)
8 months	12.2% (-1.2-24.9)	18.8% (7.9-28.9)	21.6% (11.9-31.2)	21.0% (11.7-30.6)	20.5%(11.3-29.9)
10 months	7.3% (-6.2-19.7)	14.3% (4.7-23.3)	17.5% (9.1-26.2)	16.9% (9.0-25.2)	16.3% (8.4-24.6)

OS = Overall Survival ; AE = Adverse Events ; Δ[FOLFIRINOX] = Chance of a better outcome in the FOLFIRINOX group.

Appendix Table B: Sensitivity analysis of the benefit-risk balance of FOFLIRINOX versus gemcitabine – the occurrence of a grade ≥ 3 related adverse event was analyzed as a binary outcome

Priority	Pairwise probabilities (%)		Δ[FOLFIRINOX]
	FOLFIRINOX > Gemcitabine	Gemcitabine > FOLFIRINOX	
**1 : OS (threshold = 2 months)**	54.4%	26.9%	27.4%
**2 : Worst related AE grade ≥ 3**	3.1%	5.2%	-2.1%
**Overall**	57.4%	32.1%	**25.3% (P<.001)**

OS = Overall Survival; AE = Adverse Events; Δ[FOLFIRINOX] = Chance of a better outcome in the FOLFIRINOX group.
